# Comparison of Outcomes in Dual-Mobility Versus Fixed-Bearing Implants in Primary Total Hip Arthroplasty: A Systematic Review and Meta-Analysis

**DOI:** 10.7759/cureus.61715

**Published:** 2024-06-05

**Authors:** Brandon Lim, Ariel Chai, Mohamed Shaalan

**Affiliations:** 1 School of Medicine, Trinity College Dublin, Dublin, IRL; 2 Orthopaedics and Traumatology, The Mater Misericordiae University Hospital, Dublin, IRL; 3 Trauma and Orthopaedics, St. James Hospital, Dublin, IRL

**Keywords:** tha, total hip arthroplasty complications, dual mobility, dislocation, total hip arthroplasty

## Abstract

Total hip arthroplasty (THA) is often regarded as one of the most successful surgical techniques developed in the twenty-first century. However, it is associated with complications such as prosthetic instability, dislocations, or infections. Dual-mobility (DM) implants have been developed with the goal of reducing the incidence of dislocations by increasing the femoral head-neck ratio, maximising hip stability, and improving the range of motion (ROM) before impingement and dislocation. This systematic review aims to comprehensively compare the safety and efficacy of DM versus fixed-bearing (FB) implants in primary THA patients. A comprehensive search strategy of PubMed, Embase, Scopus, and Web of Science Core Collection databases was executed to identify pertinent literature comparing DM and FB implants in THAs. Eligible studies underwent independent screening, and data were systematically extracted. The analysis employed pooled risk ratios (RR) for dichotomous outcomes and mean differences (MD) for continuous variables, each accompanied by their respective 95% confidence intervals (CI). Our systematic review and meta-analysis included nine studies encompassing 22,277 patients. The DM group had a significantly reduced incidence of dislocations compared to the FB group (RR 0.25, 95%CI [0.13, 0.47]; p-value <0.0001) and a significantly shorter length of stay (MD −9.92, 95%CI [−15.53, −4.32]; p-value = 0.0005). The FB group, however, had a significantly shorter operative time compared to the DM group (MD 10.41, 95%CI [7.64, 13.17]; p-value < 0.00001). We did not identify any significant statistical differences between the DM and FB groups regarding patient-reported outcome measures, the incidence of all-cause readmissions, the incidence of peri-prosthetic fractures, the incidence of infections, or the incidence of groyne pain.

## Introduction and background

Although total hip arthroplasty (THA) is often regarded as one of the most successful surgical techniques developed in the twenty-first century, it is also associated with complications such as prosthetic instability, dislocations, or infections [1]. The risk of dislocation is 1% within one month of surgery and 2% within one year of surgery, increasing by 1% every five years up to 7% at 25 years post-surgery [2]. Recurrent dislocations are problematic because they reduce a patient’s quality of life and may require revision surgery [3]. Instability and dislocations as a mode of failure after a THA are multifactorial problems that have been attributed to surgical factors such as surgical approach, component positioning, and component design, and patient factors such as abnormal spinopelvic mobility or spinopelvic impairment, neuromuscular disorders, cognitive dysfunction, age >75 years, abductor insufficiency, dysplastic hip, and previous surgical history [4-6]. Surgical techniques and modalities such as large prosthetic heads, trochanteric advancement, constrained liners, modular components, and dual-mobility (DM) components have thus been developed with the goal of reducing the incidence of dislocation [5].

DM implants were first developed in 1974 by Gilles Bousquet and Andre Rambert in France and feature a small femoral head articulating within a movable polyethylene liner that itself articulates within a larger acetabular shell [7]. The DM implant’s two articulations combine the principles of Charnley’s low-friction arthroplasty [8] and McKee-Farrar’s concept of hip stability [9] to increase the femoral head-neck ratio, which maximises hip stability and improves range of motion (ROM) before impingement and dislocation [10]. Anatomic dual mobility (ADM) systems and modular dual mobility (MDM) polyethylene liners were developed to increase impingement-free ROM and the effective implant head size, thereby decreasing the risk of dislocations that often arise via primary impingement between the neck of the prosthesis and articular component and secondary impingement between the bony femur and pelvis [6,11].

The benefits of DM implants compared to fixed-bearing (FB) implants were demonstrated by Stroh et al., who demonstrated dislocation rates of 0.1% and 3.5% for primary and revision DM THA, respectively, versus 2-7% and 16% for primary and revision FB THA, respectively [12]. However, the use of DM implants remains limited and is selectively used for patients with a high dislocation risk or high risk of post-operative instability, such as elderly patients, neuromuscular deficient patients, dysplastic patients, post-traumatic patients, patients with increased ligamentous laxity, patients with smaller anatomy, and patients with spinal abnormalities that reduce pelvic flexion, such as advanced degenerative spinal pathology and spinal fusion [10,13]. This hesitancy to implement a more widespread use of DM implants has been attributed to previous reports of complications such as intra-prosthetic dissociation (IPD) and accelerated wear of polyethylene acetabular liners [6,14,15].

Further investigations are thus needed to provide more comprehensive insights into the effectiveness of DM implants compared to FB implants. As such, our study aims to comprehensively compare the safety and efficacy of DM versus FB implants in primary THA patients.

## Review

Methods

Search Strategy

This study was performed according to the Preferred Items for Systematic Reviews and Meta-Analysis (PRISMA) guidelines [16]. A literature search was carried out on the PubMed, Embase, Scopus, and Web of Science Core Collection databases from their respective inceptions through April 24, 2024. The terms used were (total hip replacement OR total hip arthroplasty) AND (dual-mobility OR dual mobility) AND (fixed-bearing OR fixed bearing). Boolean operators (AND, OR) were used to combine terms and narrow search results. Reference lists of eligible articles and previous meta-analyses were examined manually to identify relevant citations.

Eligibility Criteria

During title and abstract screening, animal studies, case reports, technical reports, systematic reviews, letters to the editor, and conference abstracts were excluded. Non-comparative studies were also excluded. Studies that did not address DM versus FB implants in THA were excluded. During the full-text screening, only full-length manuscripts focused on comparing DM versus FB implants in THA were included. This process was carried out by two reviewers, and discrepancies were resolved by discussion between the two reviewers.

Study Selection

Studies were uploaded as abstracts to the online systematic review management platform Covidence (Melbourne, Australia), from which duplicate studies were automatically identified and deleted. Two reviewers evaluated the retrieved studies independently. Discrepancies were resolved by discussion between the two independent reviewers.

Data Collection

Two reviewers independently extracted study characteristics, which were recorded using Microsoft Excel sheets (Microsoft® Corp., Redmond, WA, USA). The information extracted included the author, year, country of origin, study design, number of patients, ages, gender distribution, body mass index (BMI), American Society of Anaesthesiology (ASA) score, follow-up duration, outcomes, surgical approaches, and descriptions of implants used.

The primary outcome of this review was to compare the following between primary THA with DM implants and primary THA with FB implants: (i) operative time; (ii) length of stay; (iii) patient-reported outcome measures (PROMs); (iv) incidence of all-cause readmission; (v) incidence of dislocations; (vi) incidence of infection; (vii) incidence of periprosthetic fracture; and (viii) incidence of groyne pain.

Data Analysis

Data were analysed using RevMan Web (Cochrane, London, UK). Mean differences (MD) with standard deviations were pooled for continuous outcomes. Risk ratios (RR) with 95% confidence intervals (CIs) were employed using the Mantel-Haenszel method for dichotomous outcomes. A fixed-effect model was used for homogeneous studies. A random-effects model was used for heterogeneous studies. Statistical heterogeneity was evaluated through the I2 and Chi2 tests. A p-value < 0.10 indicated heterogeneity, and I2 ≥ 50% indicated high heterogeneity.

Assessment of the Quality of Studies

Two reviewers used the Newcastle-Ottawa Scale (NOS) to assess the methodological quality and risk of bias in eligible studies [17]. This system assesses the quality of cohort studies based on selection, comparability, and outcome/exposure criteria. Scores range from zero to nine, and higher NOS scores indicate a lower risk of bias. The following variables were identified to likely confound associations between the type of implant and outcomes: (i) BMI, (ii) ASA score, and (iii) reason for primary hip replacement. Observational studies that attempted to control for one or more of these theoretical confounds were at a decreased risk of comparability bias based on NOS criteria.

Results

**Figure 1:**
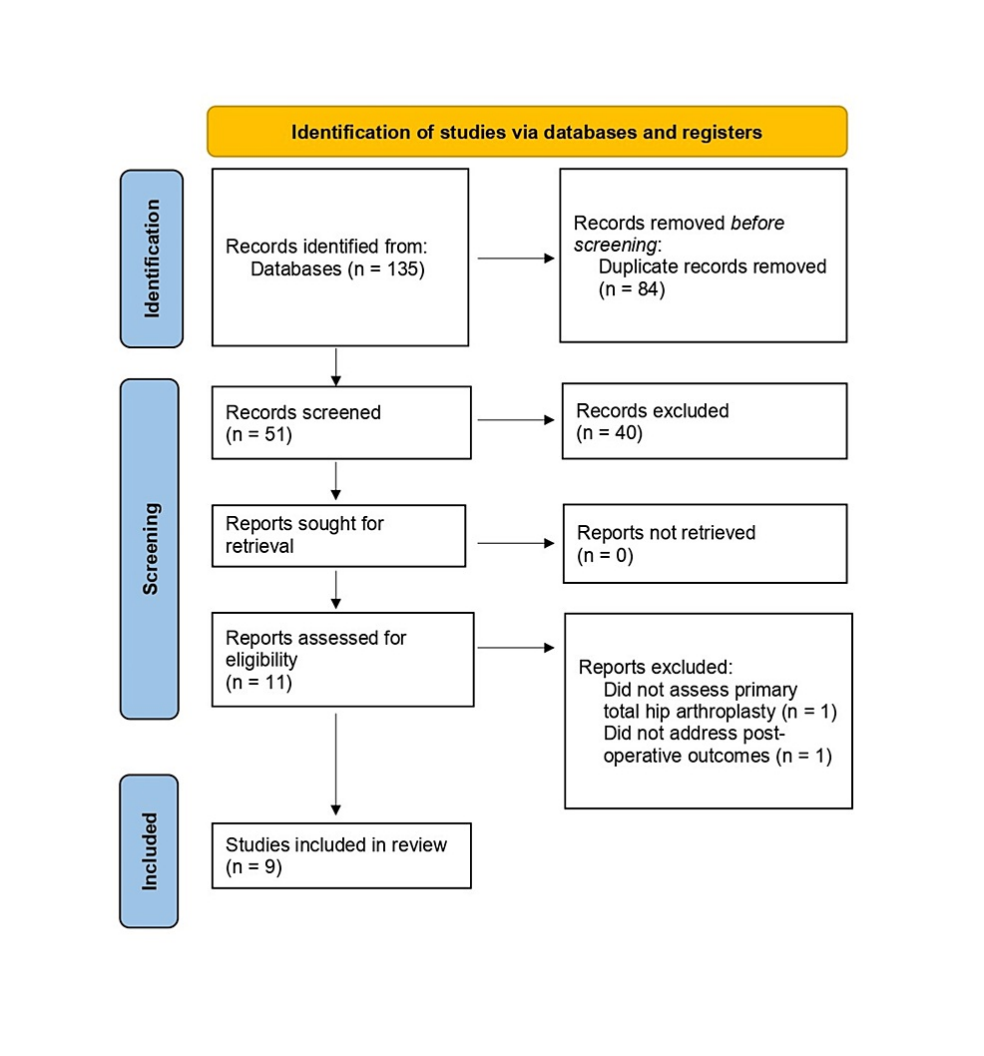
PRISMA flow chart PRISMA: Preferred Items for Systematic Reviews and Meta-Analysis

The literature search provided 135 potentially relevant articles from PubMed (n = 37), Embase (n = 35), Scopus (n = 32), and the Web of Science Core Collection (n = 31) (Figure 1). After excluding 84 duplicates, 51 records were available for title and abstract screening. Forty publications were removed after reviewing the titles and abstracts. Of the residual corpus of literature, full-text reviews were performed. This identified nine relevant studies for inclusion in this systematic review and meta-analysis.

Study Characteristics

Nine studies encompassing a total of 22,277 patients were included [10,13,18-24]. There were 2,952 total patients in the DM cohort and 19,325 total patients in the FB cohort. The mean ages in the DM group ranged from 48.5 [10] to 70.63 [22], while the mean ages in the FB group ranged from 48.4 [10] to 65.6 [19]. The mean BMI in the DM group ranged from 23.8 [20] to 32.2 [19], while the mean BMI in the FB group ranged from 24.5 [20] to 30 [13]. The ASA score was reported in four studies [13,20,23,24]. The mean ASA in the DM ranged from 1.8 [20] to 2.3 [13,24], while the mean ASA in the FB group ranged from 1.7 [20] to 2.3 [13,24]. Eight studies were retrospective [10,13,18-21,23,24] and one was prospective [22]. The characteristics of the studies selected for qualitative synthesis can be found in Table 1.

**Table 1:** Characteristics of studies selected for qualitative synthesis *Follow-up period not presented as median (range). ASA: American Society of Anaesthesiology score; DM: dual mobility; FB: fixed bearing; FJS-12: Forgotten Joint Score; HHS: Harris Hip Score; HOOSJR: Hip dysfunction and Osteoarthritis Outcome Score for Joint Replacement; IPD: intra-prosthetic dissociation; LEAS: Lower Extremity Activity Score; LOS: length of stay; MDP: Merle d'Aubigné and Postel; ROM: range of motion; SD: standard deviations; SF12: 12-Item Short Form Survey; THA: total hip arthroplasty; UCLA: University of California Los Angeles; VAS: visual analogue scale; VR-12: Veterans RAND 12 Item Health Survey; WOMAC: Western Ontario and McMaster Universities Osteoarthritis Index.

Author (year)	Origin	Study design	Study arms, n (%)	Age, mean ± SD (years)	Sex (F:M)	BMI, mean ± SD (kg/m^2^)	ASA, mean ± SD (n)	Follow-up, median (range) (years)	Primary outcomes	Secondary outcomes
Rowan et al. [10]	USA	Retrospective	DM, 136 (50)	48.5 ± 7.2	74:43	28.5 ± 6.5	-	3.2 (0.05–5.89)	Instability (including dislocation and/or IPD in the DM cohort).	Revision THA for instability.
			FB, 136 (50)	48.4 ± 6.6	79:48	28.5 ± 6.1	-	3.4 (0.1–9.1)		
Simcox et al. [13]	USA	Retrospective	DM, 508 (3.43)	59.5 ± 21.9	285:150	29 ± 7	2.3 ± 0.6	At least 90 days*	All-cause 90-day readmission and reoperation rates.	90-day readmission diagnosis and reoperation indication, surgical time.
			FB, 14,310 (96.57)	60.5 ± 23.1	7,956:6,354	30 ± 4	2.3 ± 0.6			
Moore et al. [24]	USA	Retrospective	DM, 178 (4.99)	65.8 ± 12.3	99:79	30.1 ± 6.1	2.3 ± 0.6	At least 3 months*	LOS, dislocation, anterior groyne pain, revisions, VR-12.	-
			FB, 3,390 (95.01)	63.7 ± 11.4	1,799:1,591	29.6 ± 6.2	2.3 ± 0.6			
Dubin and Westrich [18]	USA	Retrospective	DM, 664 (75.28)	61.73 ± 9.16	308:356	29.48 ± 4.92	-	2.09 (0.07–7.03)	HHS, SF12, EQ5D, LEAS.	Dislocation, readmission, revision, survivorship analysis.
			FB, 218 (24.72)	61.68 ± 9.02	102:116	29.70 ± 4.86	-	1.83 (0.10–5.40)		
Moon et al. [20]	Korea	Retrospective	DM, 63 (50)	61.6 ± 15.0	40:23	23.8 ± 4.1	1.8 ± 0.6	3.1 ± 0.7*	Dislocation	Reoperation, other various complications.
			FB, 63 (50)	60.5 ± 13.6	38:25	24.5 ± 3.3	1.7 ± 0.6	3.5 ± 1.2*		
Kim et al. [21]	Korea	Retrospective	DM, 94 (54.65)	56.70 ± 12.41	74:20	24.32 ± 3.61	-	>2 years*	Dislocation	Post-operative hip joint function.
			FB, 78 (45.35)	53.59 ± 12.57	52:26	24.63 ± 2.84	-			
Epinette [22]	France	Prospective	DM, 136 (52.11)	70.63 ± 9.498	87:49	28.448 ± 5.267	-	2–6 years*	HHS, MDP scores, THA complications, radiological assessments.	-
			FB, 125 (47.89)	65.50 ± 5.436	72:53	28.425 ± 5.436	-			
Singh et al. [23]	USA	Retrospective	DM, 298 (66.67)	64.47 ± 12.21	185:113	29.47 ± 6.84	2.2 ± 0.6	3 months, 1 year, 2 years*	All-cause 90-day readmission and reoperation rates, HOOSJR, FJS-12.	Surgical time, LOS, discharge disposition.
			FB, 149 (33.33)	65.04 ± 11.49	92:57	28.47 ± 5.85	2.2 ± 0.6			
Dubin and Westrich [19]	USA	Retrospective	DM, 875 (50.55)	66.7	342:533	32.2	-	2.8*	Groyne pain	VR-12, UCLA activity score, Pain VAS, ROM, WOMAC, revision rates.

Surgical Approach

Regarding surgical approaches, two studies utilised posterior approaches [18,19], four utilised posterolateral approaches [10,20-22], one used both posterior and anterior approaches [24], and two did not specify the surgical approach used [13,23]. Rowan et al. further elaborated that a capsular and posterior short-external rotator repair was subsequently undertaken [10]. Moore et al. specified that a posterior approach was used in 173 DM cases and 2,378 FB cases, while an anterior approach was used in 5 DM cases and 1,022 FB cases [24].

Implants

Regarding acetabular components used in DM cases, the Anatomic DM (ADM) and Modular DM (MDM) acetabular systems (Stryker Orthopaedics, Mahwah, NJ, USA) were used in five studies [10,18,19,22,23], the G7 acetabular system (Zimmer Biomet, Inc., Warsaw, IN, USA) in three studies [20,21,23], the POLARCUP (Smith and Nephew, Memphis, TN, USA) in one study [23], and the OR3O in one study (Smith and Nephew, Memphis, TN, USA) [23]. The ADM acetabular system consists of a 46-64 mm hydroxyapatite (HA)-coated press-fit acetabular cup, a 40-58 mm highly cross-linked polyethylene (HXLPE) non-constrained mobile liner, and a 28 mm constrained femoral head made of cobalt-chromium alloy (CoCr) or ceramic, while the MDM acetabular system consists of 44-80 mm HA-coated or porous titanium acetabular cups with screw holes, a highly polished modular CoCr acetabular liner, a HXLPE non-constrained liner, and a 22.2 mm or 28 mm constrained small femoral head [10]. The G7 acetabular system consists of a vitamin-E-infused, highly cross-linked polyethylene (VEPE) outer head and a delta ceramic inner femoral head [20]. The POLARCUP is made of skirted stainless steel, and the OR3O is made of zirconium [23]. Among FB cases, the Trident peripheral self-locking (PSL) acetabular system (Stryker Orthopaedics, Mahwah, NJ, USA) was used in two studies [19,22], the Trilogy acetabular system in two studies [20,21], and the G7 acetabular system in two studies [20,21]. The Trident PSL acetabular system consists of a hemispherical HA-fully coated press-fit titanium acetabular shell coupled with an HXLPE insert [22]. Prosthetic femoral heads were either made of ceramic [10,20], CoCr [10], or Oxinium [10]. Seven studies did not clearly specify the material of the femoral heads used [13,18,19,21-24]. Regarding femoral components, Kim et al. and Moon et al. used Microplasty, VerSys, and Wagner cone implants (Zimmer Biomet, Inc., Warsaw, IN, USA), with the Wagner cone being used in patients with severely narrow bone marrows [20,21]. A summary of the implants used can be found in Table 2.

**Table 2:** Summary of implants ADM: anatomic dual mobility acetabular system; CoCr: cobalt chromium alloy; DM: dual mobility; FB: fixed bearing; HXLPE: highly cross-linked polyethylene; MDM: modular dual mobility acetabular system; N/S: not specified; PE: polyethylene; PSL: peripheral self-locking; SD: standard deviations; VEPE: vitamin E-infused, highly cross-linked polyethylene

Author (year)	Study arm	Acetabular component	Acetabular liner	Femoral head	Femoral head diameter (mm)	Acetabular cup diameter (mm)	Femoral implant
Rowan et al. [10]	DM	ADM-X3 in 111 cases and MDM-X3 in 25 cases.	ADM (HXLPE), MDM (HXLPE or CoCr)	Ceramic in 74 cases, CoCr in 62 cases.	DM = 22 in 9 cases, 28 in 127 cases.	N/S	N/S
	FB	Non-cemented, press-fit titanium shells with either porous metal or HA coating.	HXLPE in 87 cases, neutral HXLPE 4 mm increased offset in 19 cases, HXLPE with elevated lip in 11 cases, ceramic in 19 cases.	Ceramic in 83 cases, CoCr in 15 cases, Oxinium in 38 cases.	FB = 28 in 13 cases, 32 in 90 cases, and 36 in 33 cases.	N/S	N/S
Simcox et al. [13]	DM	N/S	N/S	N/S	N/S	N/S	N/S
	FB	N/S	N/S	N/S	N/S	N/S	N/S
Moore et al. [24]	DM	N/S	N/S	N/S	DM = 28 in all cases. FB = 28 in 18 cases, 32 in 918 cases, 36 in 2,249 cases and 40 in 205 cases.	53 (median)	N/S
	FB	N/S	N/S	N/S		48 in 936 cases, 52 in 2,454 cases (median).	N/S
Dubin and Westrich [18]	DM	ADM or MDM.	HXLPE	N/S	N/S	N/S	N/S
	FB	Non-cemented, press-fit titanium shells with either porous metal or HA coating.	N/S	N/S	N/S	N/S	N/S
Moon et al. [20]	DM	G7 acetabular system.	3rd generation HXLPE	DM = ceramic	28 ± 0.0 (mean ± SD)	53.1 ± 3.7 (mean ± SD)	Versys Fiber Metal Taper in 7 cases, Wager SL Revision in 7, Microplasty in 49 cases.
	FB	Trilogy acetabular system in 23 cases, G7 acetabular system in 40 cases.	2nd generation HXLPE (standard) in 16 cases, 2nd generation HXLPE (elevated) in 7 cases, 3rd generation HXLPE in 40 cases.	FB = ceramic	33.0 ± 2.4 (mean ± SD)	51.8 ± 3.2 (mean ± SD)	Versys Fiber Metal Taper in 20 cases, Wager SL Revision in 7, Microplasty in 36 cases.
Kim et al. [21]	DM	G7 acetabular system.	VEPE	N/S	28 ± 0.0 (mean ± SD)	52.21 ± 3.34 (mean ± SD)	Versys, Wager Cone, or Microplasty
	FB	Trilogy or G7 acetabular systems.	PE or VEPE	N/S	33.03 ± 1.99 (mean ± SD)	51.95 ± 3.13 (mean ± SD)	
Epinette [22]	DM	Restoration ADM.	PE	N/S	28 in all cases	52.35 ± 2.84 (mean ± SD)	N/S
	FB	Trident PSL.	HXLPE	N/S	28 in all cases	52.35 ± 2.95 (mean ± SD)	N/S
Singh et al. [23]	DM	Monoblock-DM (POLARCUP or ADM), modular-DM (cylindrospheric (MDM) or subhemispheric (G7 or OR3O)).	CoCr for cylindrospheric implants, others N/S.	N/S	N/S	N/S	N/S
	FB	N/S	N/S	N/S	N/S	N/S	N/S
Dubin and Westrich [19]	DM	ADM in 584 cases, MDM in 291 cases.	N/S for ADM implants, CoCr for MDM implants.	N/S	28 (mean)	52.0 (mean)	N/S
	FB	Trident PSL.	N/S	N/S	33 (mean)	52.4 (mean)	N/S

Operative Time

**Figure 2:**
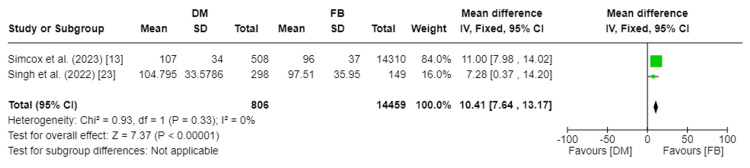
Forest plot of operative time DM: dual mobility; FB: fixed bearing

The mean operative time in minutes was assessed in two studies, including a total of 15,265 patients [13,23]. Our analysis showed a substantial difference in operative time between the DM and FB groups (MD 10.41, 95%CI [7.64, 13.17]; p-value<0.00001). Pooled studies were homogenous, so a fixed effect model was used. I2 was 0%, and Chi2-p was 0.33. Figure 2 depicts the forest plot for operative time.

Length of Stay

**Figure 3:**
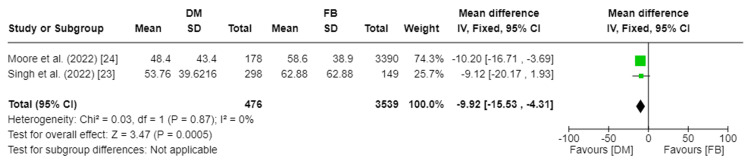
Forest plot of length of stay DM: dual mobility; FB: fixed bearing

Two studies assessed the length of stay in hours, including 4,015 patients [23,24]. Our analysis showed a substantial difference in length of stay between the DM and FB groups (MD −9.92, 95%CI [−15.53, −4.32]; p-value = 0.0005). Pooled studies were homogenous, so a fixed-effect model was used. I2 was 0%, and Chi2-p was 0.87. Figure 3 depicts the forest plot for the length of the stay.

All-Cause Readmissions

**Figure 4:**
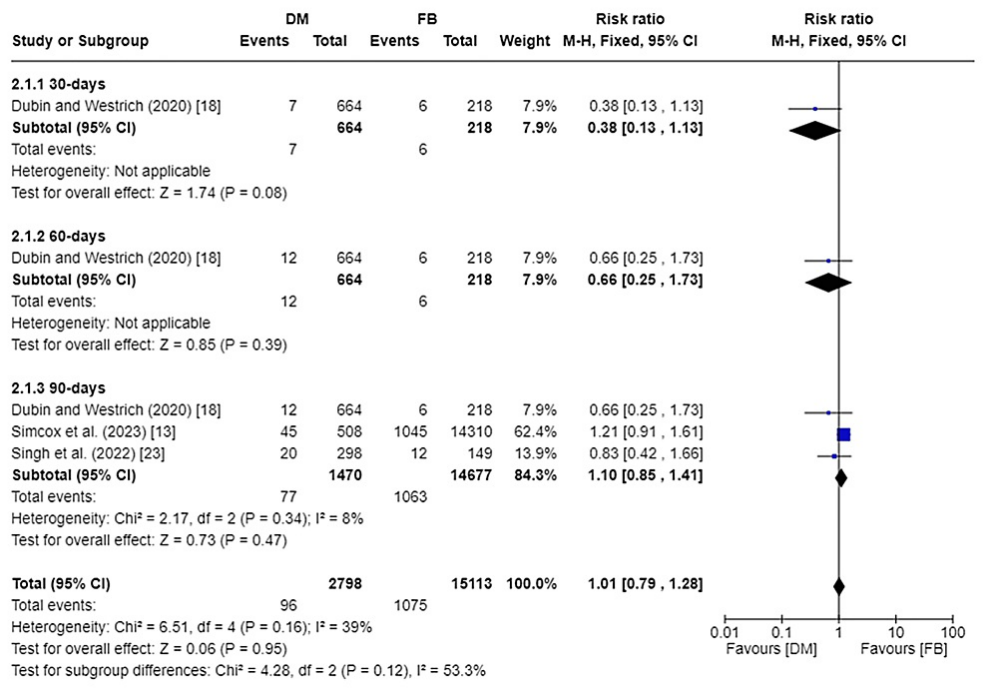
Forest plot of all-cause readmissions DM: dual mobility; FB: fixed bearing

All-cause readmissions were assessed in three studies [13,18,23]. At 30 days, all-cause readmissions were only assessed in one study encompassing 882 patients [18]. There was no significant statistical difference in the incidence of 30-day readmission between DM and FB groups (RR 0.38, 95%CI [0.13, 1.13]; p-value = 0.08). At 60 days, all-cause readmissions were only assessed in one study encompassing 882 patients [18]. There was no significant statistical difference in the incidence of 60-day readmission between the DM and FB groups (RR 0.66, 95%CI [0.25, 1.73]; p-value = 0.39). At 90 months, all-cause readmissions were assessed in three studies, encompassing a total of 16,147 patients [13,18,23]. There was no significant statistical difference in the incidence of 90-day readmission between the DM and FB groups (RR 1.10, 95%CI [0.85, 1.41]; p-value = 0.47). Pooled studies reporting 90-day all-cause readmissions were homogenous, so a fixed effect model was used. I2 was 8%, and Chi2-p was 0.34. Figure 4 depicts the forest plot for all-cause readmissions.

Patient-Reported Outcome Measures

PROMs were reported in seven studies [10,18-23]. These include the Harris Hip Score (HHS) [18,22], the modified HHS (mHHS) [10,20,21], the Veterans RAND 12 Item Health Survey (VR-12 score) [19,24], the Hip Dysfunction and Osteoarthritis Outcome Score for Joint Replacement (HOOSJR) score [23], the Forgotten Joint Score (FJS-12) score [23], Merle d'Aubigné-Postel (MDP) score [22], the University of California Los Angeles (UCLA) score [19], Western Ontario and McMaster Universities Osteoarthritis (WOMAC) index [19], Visual Analogue Scale (VAS) Pain [19], the SF12 score [18], the EQ5D score [18], and the Lower Extremity Activity Score (LEAS) [18].

**Figure 5:**
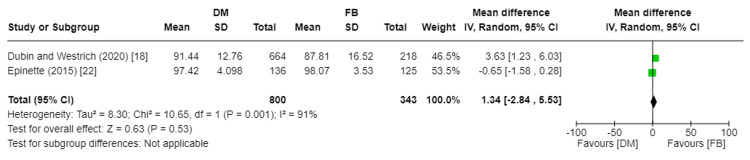
Forest plot of Harris Hip Score (HHS) DM: dual mobility; FB: fixed bearing

HHS was reported in two studies encompassing 1,143 total patients [18,22]. Epinette recorded HHS at a minimum of two years of follow-up [22], while Dubin et al. recorded HHS at a mean of 2.09 years and 1.83 years in the DM and FB groups, respectively [18]. There was no significant statistical difference in HHS between the DM and FB groups (MD 1.34, 95%CI [−2.84, 5.53]; p-value = 0.53). The pooled studies reporting HHS were heterogenous, indicating the complexity of the research. A random effect model was used, with I2 at 91% and Chi2-p at 0.001. Figure 5 depicts the forest plot for HHS, further illustrating the heterogeneity in the studies.

**Figure 6:**
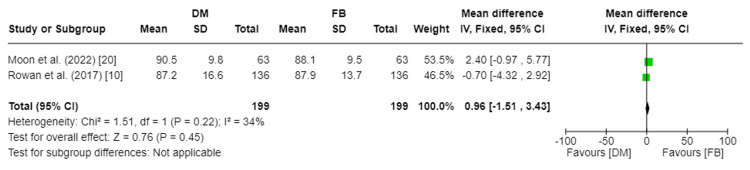
Forest plot of modified Harris Hip Score (mHHS) DM: dual mobility; FB: fixed bearing

mHHS was reported in three studies encompassing 570 total patients [10,20,21]. Rowan et al. recorded mHHS at a mean of 3.2 years and 3.4 years in the DM and FB groups, respectively [10]. Kim et al. recorded mHHS pre-operatively, post-operatively, and at one year and two years for both groups [21], and Moon et al. recorded mHHS at a mean of 3.1 years and 3.5 years in the DM and FB groups, respectively [20]. The studies by Rowan et al. and Moon et al. were meta-analysed, given that the mean follow-up at which mHHS was recorded was >3 years. There was no significant statistical difference in mHHS between the DM and FB groups (MD = 0.96, 95%CI [−4.32, 2.92]; p-value = 0.45). Pooled studies reporting mHHS were homogenous, so a fixed effect model was used. I2 was 34%, and Chi2-p was 0.22. Figure 6 depicts the forest plot for mHHS.

VR-12 scores were reported by two studies encompassing 5,299 total patients [19,24]. Moore et al. recorded VR-12 scores pre-operatively, at three months, and at one year [24], while Dubin and Westrich recorded VR-12 scores at a mean of 2.8 and 3.1 years in the DM and FB groups, respectively [19]. We did not carry out a meta-analysis for these two studies, given the difference in their follow-up times.

Incidence of Dislocations

**Figure 7:**
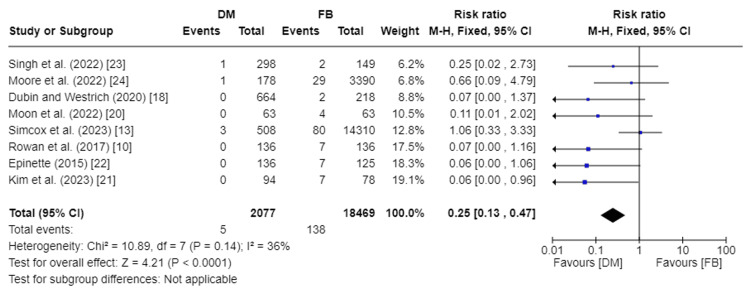
Forest plot of incidence of dislocations DM: dual mobility; FB: fixed bearing

The incidence of dislocations was recorded in eight studies, encompassing 20,546 total patients [10,13,18,20-24]. Our analysis showed a significant statistical difference in the incidence of dislocations between DM and FB groups (RR 0.25, 95%CI [0.13, 0.47]; p-value <0.0001). Pooled studies were homogenous, so a fixed effect model was used. I2 was 36%, and Chi2-p was 0.14. Figure 7 depicts the forest plot for the incidence of dislocations.

Incidence of Peri-Prosthetic Fractures

**Figure 8:**
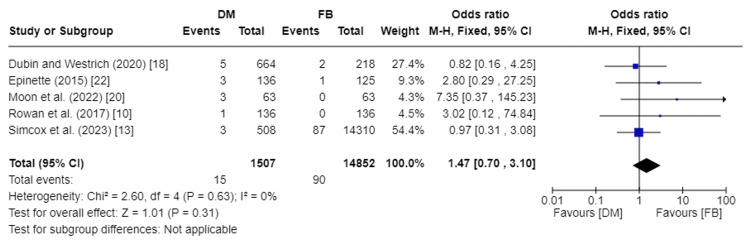
Forest plot of incidence of peri-prosthetic fractures DM: dual mobility; FB: fixed bearing

The incidence of peri-prosthetic fractures was recorded in five studies, encompassing 16,359 total patients [10,13,18,20,22]. Our analysis showed no significant statistical difference in the incidence of peri-prosthetic fractures between DM and FB groups (RR 1.47, 95%CI [0.70, 3.10]; p-value = 0.31). Pooled studies were homogenous, so a fixed effect model was used. I2 was 0% and Chi2-p was 0.63. Figure 8 depicts the forest plot for incidence of peri-prosthetic fractures.

Infections

**Figure 9:**
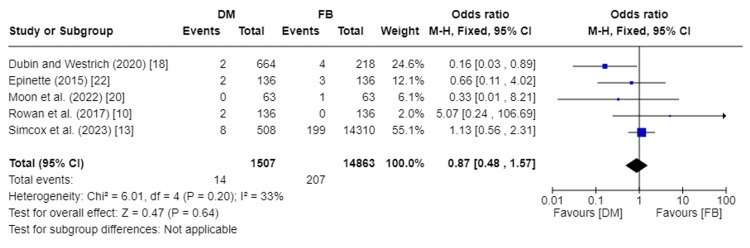
Forest plot of incidence of infections DM: dual mobility; FB: fixed bearing

The incidence of infections was recorded in five studies, encompassing 16,359 total patients [10,13,18,20,22]. Our analysis showed no significant statistical difference in the incidence of infections between DM and FB groups (RR 0.87, 95%CI [0.48, 1.57]; p-value = 0.64). Pooled studies were homogenous, so a fixed effect model was used. I2 was 33%, and Chi2-p was 0.20. Figure 9 depicts the forest plot for the incidence of infections.

Groin Pain

**Figure 10:**
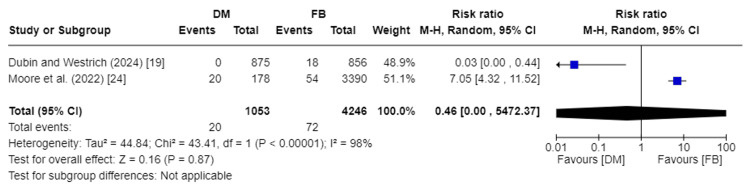
Forest plot of groin pain DM: dual mobility; FB: fixed bearing

Groin pain was evaluated in two studies encompassing 5,299 total patients [19,24]. Our analysis showed no substantial difference in the incidence of groin pain between the DM and FB groups (RR 0.46, 95%CI [0.00, 5472.37]; p-value = 0.87). Pooled studies were heterogeneous, so a random effect model was used. I2 was 98%, and Chi2-p was <0.00001. Figure 10 depicts the forest plot for groin pain.

Quality of Studies

NOS scores ranged from seven [10,13,18,20-24] to eight [19] (Table 3). Regarding selection bias, all nine studies were representative of the average patient with a DM implant THA in the community, selected patients with FB implants from the same communities, and ascertained exposures through secure records or structured interviews [10,13,18-24]. Since these studies compared post-operative outcomes between DM and FB implant groups, outcomes could not have been measured or demonstrated at the start of the studies. Regarding comparability bias, all nine studies controlled for BMI were included [10,13,18-24] and four studies controlled for ASA score [13,20,23,24]. Regarding outcome bias, all nine studies confirmed outcomes using secure medical records [10,13,18-24]. A follow-up time of three months was deemed adequate for the outcome of interest to occur. This time frame was met in all nine studies [10,13,18-24]. Lastly, a loss of <20% was deemed acceptable. Dubin and Westrich [19] reported that 43 (4.3%) of patients in the DM cohort and 65 (7.8%) patients in the FB cohort were lost to follow-up [19]. The other eight studies did not comment about losses to follow-up [10,13,18,20-24].

**Table 3:** Newcastle-Ottawa Scale Scores NOS: Newcastle-Ottawa Scale

NOS items	Rowan et al. [10]	Simcox et al. [13]	Moore et al. [24]	Dubin and Westrich [18]	Moon et al. [20]	Kim et al. [21]	Epinette [22]	Singh et al. [23]	Dubin and Westrich [19]
Representativeness of the exposed cohort	1	1	1	1	1	1	1	1	1
Selection of the non-exposed cohort	1	1	1	1	1	1	1	1	1
Ascertainment of exposure	1	1	1	1	1	1	1	1	1
Demonstration that the outcome of interest was not present at the start of the study									
Comparability of cohorts on the basis of the design and analysis	2	2	2	2	2	2	2	2	2
Assessment of outcome	1	1	1	1	1	1	1	1	1
Was follow-up long enough for outcomes to occur	1	1	1	1	1	1	1	1	1
Adequacy of follow-up of cohorts									1
Total (out of 9)	7	7	7	7	7	7	7	7	8

Discussion

This systematic review and meta-analysis aimed to compare the outcomes between DM implants and FB implants used in THA. Nine studies, encompassing a total of 22,277 patients, were included in the final analysis. The DM group had a significantly reduced incidence of dislocations compared to the FB group (p-value <0.0001) and a significantly shorter LOS (p-value = 0.0005). The FB group, however, had a significantly shorter operative time compared to the DM group (<0.00001). We did not identify any significant statistical differences between the DM and FB groups regarding HHS and mHHS, the incidence of all-cause readmissions, the incidence of peri-prosthetic fractures, the incidence of infections, or the incidence of groyne pain.

The two distinct articulations in DM implants were introduced with the aim of decreasing the risk of dislocation after a THA by using a larger effective head diameter, allowing for a greater range of motion before complete dislocation can occur, increasing stability, and reducing impingement between the neck and cup rim [25]. The results of our study demonstrated this reduced risk of dislocation in DM implants compared to FB implants. These findings also align with previous literature [25-27]. A study by Harwin et al. comparing outcomes of DM implants versus FB implants in revision THA found one dislocation among 85 patients who underwent revision THA using DM implants and six dislocations among 170 patients who underwent revision THA using FB implants (1.18% versus 3.53%) [27]. A previous systematic review and meta-analysis by Romagnoli et al. encompassing 2,408 patients and comparing the efficacy of DM implants in preventing dislocation after THA compared to FB implants concluded that DM implants resulted in lower incidences of dislocations (RR 0.16, 95%CI [0.09, 0.28]; p-value <0.00001) [25]. Another systematic review by Zampogna et al., encompassing 1,530 patients and analysing the outcomes and complications of DM implants in patients younger than 55 years old, reported 46 (2.7%) cases of dislocations where the PE liner dissociated from the femoral head [26]. Lastly, although patients with hip dysplasia have a high risk of dislocation due to anatomical deformities, DM implants have been found to lower the risk of post-operative dislocations [21].

Regarding mean operative time, our analysis showed a substantial difference in operative time between the DM and FB groups. Simcox et al. attributed this to the time required to assemble the DM implant and surgeons utilising a DM implant after failing to achieve desired stability with a trial FB implant [13].

Among the seven studies [10,18-23] that reported PROMs, 12 different scores were used. Our study did not identify any significant statistical differences between the DM and FB groups regarding HHS and mHHS. Similarly, a study of DM implants in revision THAs by Harwin et al. also did not find any statistically significant improvement in pre-operative and post-operative HHS scores [27]. Conversely, a systematic review of DM implants in patients younger than 55 years old by Zampogna et al. found that the mean value of the HHS score increased from 50.9 pre-operatively to 91.6 post-operatively [26]. This may suggest that DM implants may be more well tolerated in young patients undergoing a primary THA compared to patients undergoing a revision THA.

Regarding other post-operative complications, we found no significant statistical difference between the DM and FB groups regarding the incidence of all-cause readmissions, the incidence of peri-prosthetic fractures, the incidence of infections, or the incidence of groyne pain. In addition to these complications that were analysed in this study, other complications that may arise from a THA include venous thromboembolism [13,20], pneumonia [20], urinary tract infections [20], enteritis with ileus [20], rheumatoid flares [20], and post-operative anaemia [13]. Other complications of DM implants that must be noted are intra-prosthetic dissociation (IPD), which can lead to wear, metallisation, and collision of the femoral head [21].

In addition to better outcomes when using DM implants versus FB implants, DM implants also appear to be more cost-effective than FB implants [28]. A medicoeconomic study in France carried out by Epinette et al. found that, when comparing the costs between the two types of implants in a cohort of 100,000 patients while assuming a relative risk of dislocation of 0.4 with DM implants versus FB implants, DM implants avoid 3,283 dislocations, 882 revisions, and 932 rehabilitation unit admissions while potentially saving 28.3 million euros per year for 100,000 THAs performed annually and 283 euros per patient [28]. An analysis of 235,857 revision THAs by Bozic et al. [1] reported that revision THAs had an average cost of $23,130 ± 36,643 per hospitalisation and a higher length of stay for revision THAs (mean ± SD, 5.8 days ± 14.0 days) than revision TKAs (mean ± SD, 4.8 days ± 10.5 days). Therefore, the cost-effectiveness of DM implants should be considered when choosing between DM and FB implants for a THA.

This study was not without limitations. The literature search utilised four databases (PubMed, Embase, Scopus, and Web of Science Core Collection), which may not have included all relevant studies that may have been available on other databases. The search was limited to articles in English and grey literature was not examined, so relevant articles that fall under these categories would have been excluded. Confounding factors in baseline characteristics such as BMI and ASA scores across studies could have influenced observed outcomes. Sample sizes between studies also ranged from 126 [20] to 14,818 [13]. Simcox et al. also featured unequal arms (DM vs. FB) with a substantial ratio disparity (1:28), which could introduce bias and impact the meta-analysis [13]. Follow-up times also ranged from three months [13,24] to six years [22], which may have influenced the comparative analysis given that there is no fixed timing at which post-operative complications can arise.

## Conclusions

Our review showed that patients who had undergone a primary THA with DM implants had a significantly reduced incidence of dislocations and a significantly shorter length of hospital stay compared to those who had received FB implants. However, THAs that use FB implants have a significantly shorter operative time. There were no significant differences in PROMs such as the HHS and mHHS, the incidence of all-cause readmissions, the incidence of peri-prosthetic fractures, the incidence of infections, or the incidence of groyne pain. DM implants also appear to be more cost-effective than FB implants. These factors that we have discussed in our review should, therefore, be considered and evaluated when choosing between DM and FB implants for a primary THA.
